# Identification of Immune-Related Hub Genes in Thymoma: Defects in CD247 and Characteristics of Paraneoplastic Syndrome

**DOI:** 10.3389/fgene.2022.895587

**Published:** 2022-06-14

**Authors:** Lin-Fang Deng

**Affiliations:** ^1^ College of Sciences, Shanghai University, Shanghai, China; ^2^ College of Medicine, Shanghai University, Shanghai, China

**Keywords:** Thymomas, immune, WGCNA, paraneoplastic syndrome (PNS), CD247

## Abstract

**Background:** Thymomas (Ts) and thymic carcinomas (TCs) are rare primary tumors of the mediastinum. Paraneoplastic syndrome (PNS) is an important feature of thymoma, which presents great challenges to clinicians.

**Methods:** The present study uses the weighted gene co-expression network analysis (WGCNA) to identify possible immunologic mechanisms of thymoma. RNA sequencing data from thymoma samples were downloaded from the TCGA. Core genes were taken from the module that is closely related to the WHO’s stage of classification. Enhanced analysis using the online database “Metascape” and an overall survival (OS) analysis were carried out via the Kaplan–Meier method. The hub genes were obtained from the protein–protein interaction (PPI) network. In addition, we jointly analyzed multiple sets of PNS data related to thymomas from other sources to verify the correlation between thymomas and PNS. The impact of hub genes on the prognosis of PNS was evaluated via the ROC curve, with simultaneous analysis of immune infiltration by CIBERSORT.

**Findings:** The 14 immune hub genes closely related to thymomas were found to be jointly involved in the T-cell receptor signaling pathway. Compared to the normal thymus and type B1/B2 thymoma, there is a lower number of T-cells in type A/B3 thymoma and thymic carcinoma. The expression of genes related to the T-cell receptor signaling pathway appeared defective. The low expression of CD247 and the decrease in the number of mature T-cells are common features among thymomas, specific pulmonary fibrosis, rheumatoid arthritis, and systemic lupus erythematosus.

## Introduction

Thymomas (Ts) and thymic carcinomas (TCs) are rare primary tumors of the mediastinum, originating from the thymic epithelium (Scorsetti et al., 2016). The World Health Organization (WHO) divides thymoma into type A, AB, B1, B2, B3, and C according to the morphology of thymoma epithelial cells and the ratio of lymphocytes to epithelial cells in tissues (Marx et al., 2015; Travis et al., 2015). Type C is a thymic carcinoma, and others are thymomas. Thymomas are relatively common primary anterior mediastinal mass, while thymic carcinoma is rare. These two can be synchronous. Thymoma sometimes can develop into carcinomas, but it usually takes 10 to 14 years (Ettinger et al., 2013). In addition to the WHO classification system, the Masaoka staging system and TNM staging system are also commonly used in clinical thymoma (Ried et al., 2018). Masaoka classifies thymomas into stages I, II (IIa and IIb), III, and IV (IVa and IVb) according to their comprehensive capsule infiltration and tumor metastasis (Masaoka, 2010). However, the TNM staging system includes lymph nodes in the evaluation criteria, which is of great significance for thymoma. In general, there appears to be a clear correlation and correspondence between several staging methods.

Due to the insidious onset and slow progression of thymoma, the first diagnosis is usually found by chance in physical examination, or physical discomfort caused by paraneoplastic syndrome (PNS). PNS is an important feature of thymoma, bringing great challenges to clinicians (Blum et al., 2020). The common PNS of thymoma are myasthenia gravis, total red cell aplasia, polymyositis, systemic lupus erythematosus, rheumatoid arthritis, Cushing syndrome, and syndrome of inappropriate antidiuretic hormone secretion (Rajan and Zhao, 2019; Rubinstein et al., 2019; Hashimoto et al., 2020; Sideris and Huang, 2020). A sizeable percentage (25–40%) of thymoma patients with myasthenia gravis, and more than 15% of patients with fever and immunodeficiency such as syndromes (Zhao et al., 2020; Liao et al., 2021). Thus, deciphering the relationship between this particular clinical symptom and tumor is crucial for the treatment of thymoma. The treatment and management of thymomas include chemoradiotherapy, the use of corticosteroids, immunotherapy, tyrosine kinase inhibitors, and surgical resection. However, the complications, caused by surgery and radiotherapy, such as radiation pericarditis, radiation pneumonia, and pulmonary fibrosis can hasten the patients’ death (Ettinger et al., 2013; Jeffrey Yang et al., 2020; Tian et al., 2020; Yu et al., 2020).

In recent years, with the development of systems biology, an increasing number of researchers have explored and predicted the prognostic targets of tumors from the perspectives of gene expression and molecular interaction. However, the etiology of thymoma is still unclear, and the association between PNS and thymoma is also ambiguous. Therefore, this study explores the biochemical mechanism of thymoma from the perspective of systems biology. Weighted correlation network analysis (WGCNA) is a high-quality method for finding clusters (modules) of highly correlated genes (Langfelder and Horvath, 2008; Langfelder and Horvath, 2012), which is a method of data reduction and unsupervised classification. This method can form a module of co-expressed genes to simplify the complex data matrix. Moreover, we can further explore the correlation between gene network and phenotype of concern and explore the hub genes in the network. This method has been widely used since its development. For example, Tian et al. used WGCNA to identify two gene co-expression modules involved in the process of lung squamous cell carcinoma metastasis and suggested that CFTR, SCTR, and FIGF genes could be used as a potential prognostic biomarker (Tian et al., 2017). Magdalena Niemira et al. (2019) applied WGCNA for exploring molecular networks associated with clinical traits such as tumor size, SUV max, BMI, smoking status, recurrence-free survival, and disease-free survival (Niemira et al., 2019). In addition, WGCNA has been applied to breast cancer, liver cancer, colon adenocarcinoma, and other diseases (Yin et al., 2018; Zhou et al., 2018; Wang et al., 2019).

This study analyzes the immunologic mechanism of thymoma by WGCNA. RNA sequencing data from thymoma samples were downloaded from the TCGA. The thymomas datasets GSE177522, GSE57892, and GSE29695 from GEO were used as the validation set to verify the reliability of the results. In addition, we jointly analyze multiple sets of PNS data related to thymomas (GSE33566, GSE93272, and GSE138458) to explore the internal relationship between thymomas and PNS.

## Materials and Methods

### Data Sources and Pre-Processing

RNA sequencing data and clinical information for thymomas were downloaded using TCGAbiolinks, a third-party tool officially recommended by GDC in R language (Colaprico et al., 2016; Silva et al., 2016; Mounir et al., 2019). The TCGAbiolinks tool downloads data through GDC official API. There were 119 cases of thymomas (including 36 cases of type AB thymoma, 15 of type A thymoma, 14 of type B1 thymoma, 30 of type B2 thymoma, 13 of type B3 thymoma, and 11 of thymic carcinomas) and two cases of normal thymus tissue. The validation sets GSE177522, GSE57892, and GSE29695 were downloaded from the GEO official website (https://www.ncbi.nlm.nih.gov/geo/). Some common PNS data were also downloaded from the GEO official website, including rheumatoid arthritis (GSE93272), systemic lupus erythematosus (GSE138458), and pulmonary fibrosis (GSE33566).

**FIGURE 1:**
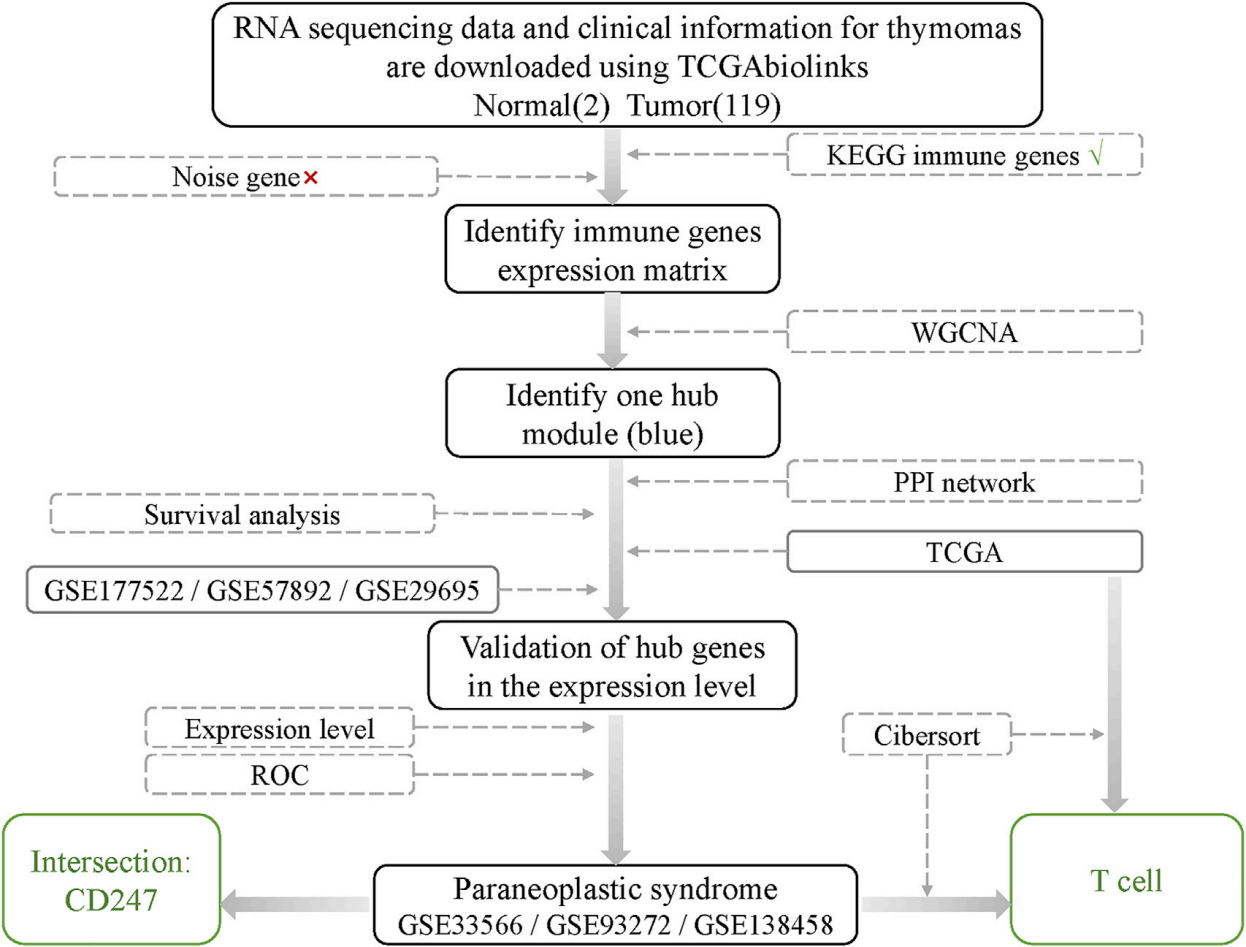
Workflow of searching hub genes in thymoma.

The workflow of the study is shown in Figure 1.

### Screening of Immune Gene and Noise Gene

The purpose of this study is to find out the relationship between thymoma and immunity. Therefore, a total of 2,381 genes in all immune-related pathways were collected from the KEGG website (https://www.genome.jp/kegg/). First, we extract the expression profiles of these immune genes from thymoma data. For details of the gene list, cf. Table 1.XLSX of the Supplementary Materials (SM). Second, noise genes were screened by the correlation method. Specifically, the Spearman correlation coefficient matrix of immune genes was calculated. It was determined that the two genes are not correlated when the correlation coefficient r is in the range of (−0.2, 0.2). When a gene is not correlated with 70 % of the remaining genes, it is determined to be a noise gene (cf. SI-1 of the Data Sheet 1.PDF). The data matrix of the optimized immune-related genes is 2023 * 121.

### Weighted Gene Co-Expression Network Analysis

This study uses WGCNA (version: 1.70–3), downloaded by BiocManager (version: 1.30.10) in the R suite, to construct the immune co-expression network (Langfelder and Horvath, 2008; Langfelder and Horvath, 2012). After screening and testing the immune expression matrix, the Pearson correlation coefficient matrix is calculated and a suitable soft threshold is selected.

$$c_{ij}=\frac{cov\left(i,j\right)}{\sigma_{i}\sigma_{j}},$$

where cov(i,j) refers to the covariance of genes i and j , σ represents the standard deviation of gene, and $c_{ij}\;$ is the Pearson correlation coefficient between genes i and j . Then, the adjacency matrix, a matrix of weighted correlation between genes, was constructed using the power function. This method strengthens the strong correlation and weakens the weak correlation or negative correlation, which makes the correlation value more in line with the scale-free network characteristics and more biological significance.

$$a_{ij}={\left|c_{ij}\right|}^{\beta },$$

where $a_{ij}$ is adjacency between those two genes. Then, the topological overlap matrix (TOM) was constructed using the adjacency function to reduce noise and false correlation.

$$TOM_{i,j}=\frac{I_{ij}+a_{ij}}{min\left(k_{i}+k_{j}\right)+1-a_{ij}},$$

where $I_{ij}$ is the product’s sum of the adjacency coefficients of the nodes connected by genes i and j , and k refers to the sum of the adjacency coefficients of the given gene with all other genes in the weighted network. The TOM is a method to quantitatively describe the similarity in nodes by comparing the weighted correlation between two nodes and other nodes.

### Identification of Clinically Significant Modules and Immune Hub Genes in Thymoma

The co-expression module is a collection of genes with high topological overlap similarity. First, principal component analysis (PCA) was used to find the first principal component of each module (module eigengene, ME) to represent the expression pattern of the module. Then, the correlation between these modules and clinical data was calculated to determine the concerned clinical information and gene modules. Module membership (MM) and gene significance (GS) were used to describe the reliability of a gene in the module. The intramodular connectivity may be interpreted as a measure of MM. Genes with intramodular connectivity greater than 0.8 were selected as hub genes (highly connected genes).

### Survival Analysis of Hub Genes

The ability of hub genes to predict survival is based on Kaplan–Meier analysis by using the “survival” package (version: 3.2–7) in the R suite. First, the expression profile of hub genes was extracted from TCGA data. Second, the median expression value of each hub gene was determined. Third, the tumor samples were divided into high-expression groups and low-expression groups with the median of each gene as the boundary. The median sample is divided into high-expression groups. Finally, differences in survival between high- or low-expression groups were assessed for significance by the log-rank test. If p<0.05 , we consider the gene to be a validated hub gene.

### Functional Enrichment of Hub Genes

In order to analyze the biological functions and signaling pathways of differentially expressed genes in thymoma-related modules, we perform enrichment analysis using the online database “Metascape” (https://metascape.org/gp/index.html#/main/step1) (Zhou et al., 2019). At the same time, protein–protein interaction (PPI) network can be given by Metascape, which uses the molecular complex detection (MCODE) algorithm.

### CIBERSORT

CIBERSORT (Newman et al., 2015) is an R package/webpage tool for deconvolution of the expression matrix of human immune cell subtypes based on the principle of linear support vector regression. The proportions of the 22 tumor-infiltrating immune cells (TIICs) from each sample were determined by using the “CIBERSORT” (R package). CIBERSORT was used to analyze the relative expression levels of 547 genes in individual tissue samples according to their gene expression profiles, to predict the proportion of 22 types of TIICs in each tissue. CIBERSORT derived a *p*-value for the deconvolution of each sample, which provided a measure of confidence in the results, and p < 0.05 was considered accurate. Significant results ( p < 0.05 ) were selected for subsequent analysis.

The CIBERSORT results of TCGA thymoma data were downloaded from the GDC website (https://gdc.cancer.gov/about-data/publications/panimmune) (Thorsson et al., 2018). The CIBERSORT result of GEO data was computed using the “cibersort” package of R (the number of permutations: perm =1,000). For details of the list, see Supplementary Material: *results_cibersort.xlsx*.

## Results

### Immune Gene Expression Profile Data in Thymoma

**FIGURE 2:**
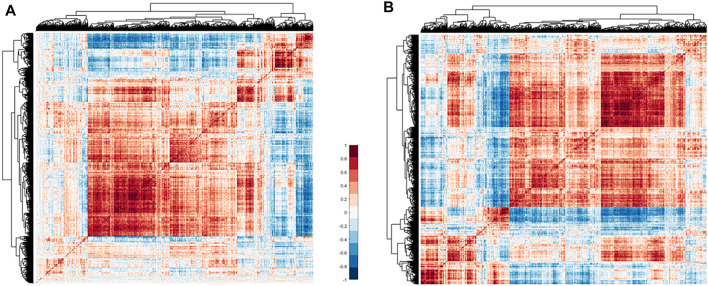
Heat map of immune gene correlation matrix clustering. **(A)** Correlation matrix clustering hot map of 2,247 immune genes in TCGA thymoma data. **(B)** The 2,023 immune gene correlation matrix clustering heat map. Of these, 224 noise genes are eliminated by the correlation method. Red means positive correlation, blue means negative correlation, and white means no correlation.

A total of 2,381 genes related to immune pathways were obtained from the KEGG website. The data matrix of the immune gene expression profile of thymoma was obtained by taking intersection with TCGA thymoma expression profile data, and the matrix dimension was 2247 ∗ 121 . Then the noise genes were screened by the correlation method, and the optimized immune gene expression data matrix of thymoma was obtained, with the matrix dimension of 2023 ∗ 121 . The optimized matrix avoided the interference of noise in the analysis, and there was a strong correlation between the 2,023 immune genes that remained. The correlation heat map of the immune expression data before and after noise reduction is shown in Figure 2.

### Construction of Weighted Gene Co-Expression Network

WGCNA was used to construct a network based on the expression matrix of 2023 immune genes and clinical data from 121 thymoma samples.

**FIGURE 3:**
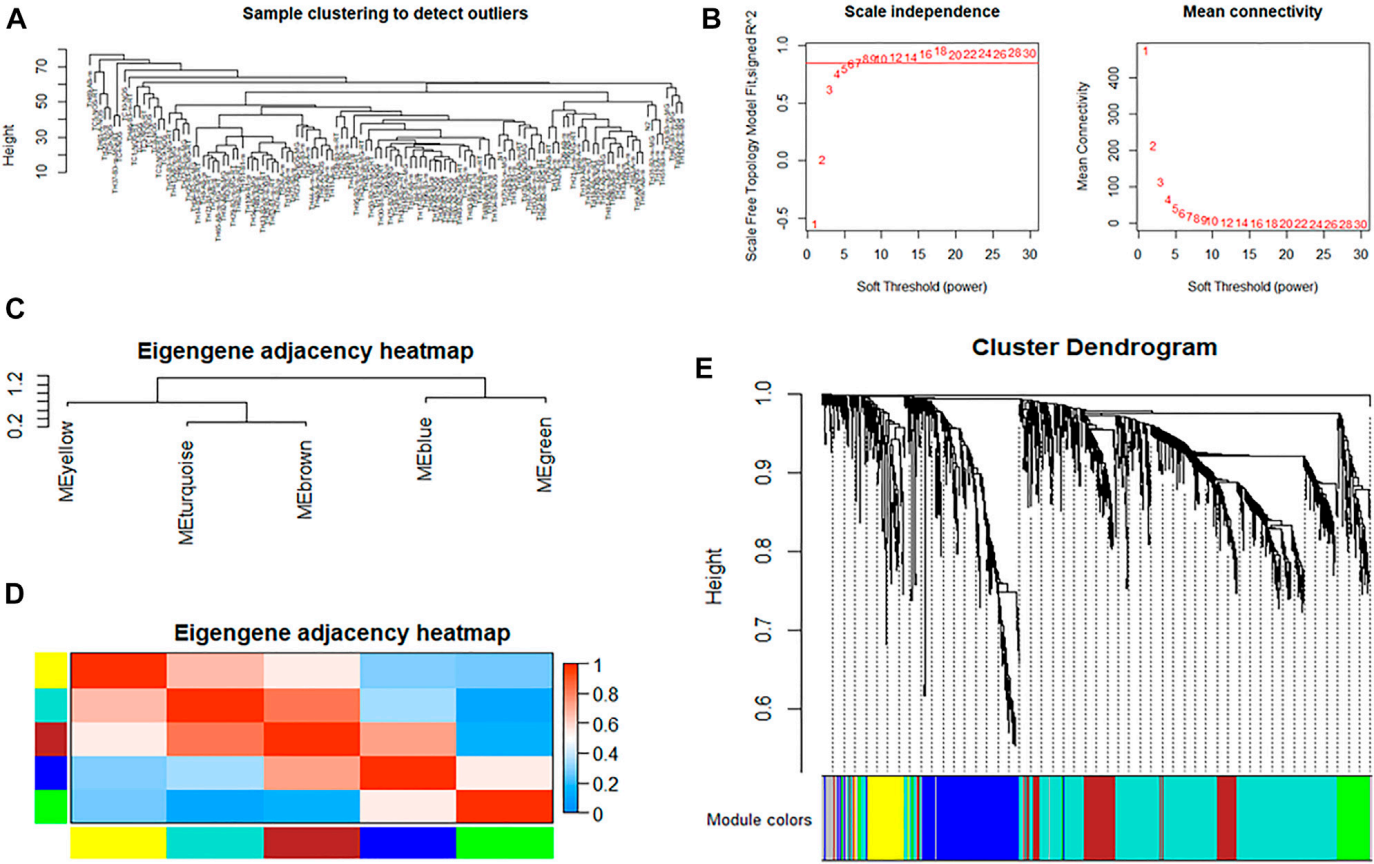
WGCNA of immune genes in thymoma. **(A)** Cluster analysis of samples to detect outliers. **(B)** Determination of soft-thresholding power in weighted gene co-expression network analysis. The left shows the scale-free fit index (*y*-axis) as a function of the soft-thresholding power (*x*-axis). The right shows the average connectivity (degree, *y*-axis) as a function of the soft-thresholding power (*x*-axis). **(C,D)** Module eigengene dendrogram and heatmap of eigengene adjacency. **(E)** Clustering dendrogram of genes, with dissimilarity based on the topological overlap, together with assigned module colors.

A dendrogram of samples was clustered by the average linkage method and Pearson’s correlation method to check the quality of the data from the 121 samples, and no outliers were identified for removal (Figure 3A). To construct a scale-free network, we set the soft threshold power β to 8, and the independence degree to 0.9 (Figure 3B). The result showed that there were a total of five co-expression modules; the gray module contains genes that could not be incorporated into any other module (Figures 3C–E).

**FIGURE 4:**
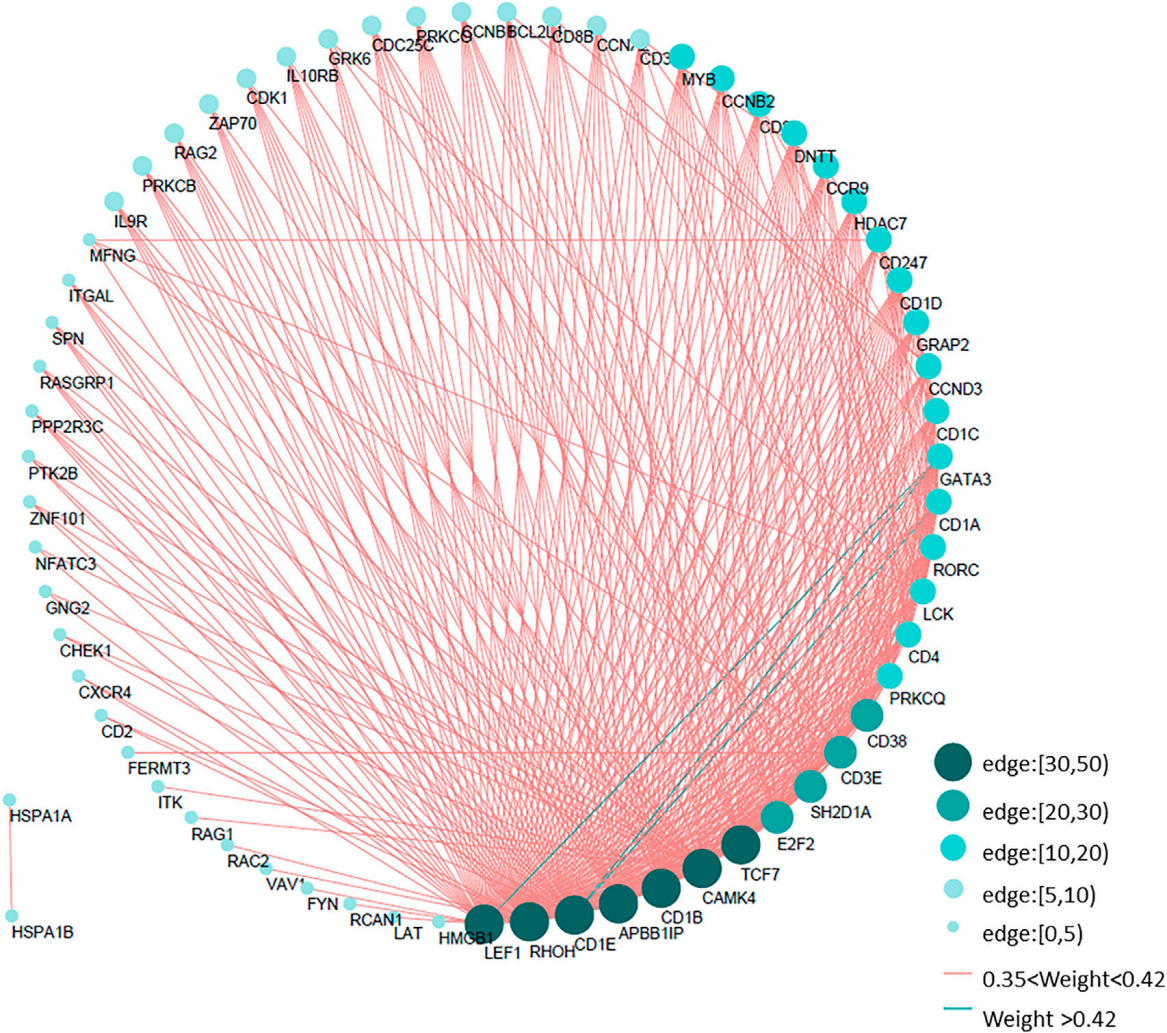
Immune gene interaction network of thymoma. According to the gene interaction table obtained by WGCNA, the edges with a weight greater than 0.35 in five modules are selected to draw the immune network of thymoma. The size of the circle and the depth of the color indicate the different number of edges, cf. Table 3.XLSX of the Supplementary Material.

According to the topological overlap matrix (TOM), the connection relationship between genes in each module could be obtained. Different colors indicate that the weight in different modules was greater than 0.35, and the immune gene interaction network of thymoma was visualized by Cytoscape. Among them, LEF1, RHOH, APBB1IP, CD1B, CAMK4, and TCF7 interact with more than 30 immune genes, which are the core genes of the immune network of thymoma, shown as Figure 4.

### Association of Modules With Clinical Traits and Determination of Core Genes

**FIGURE 5:**
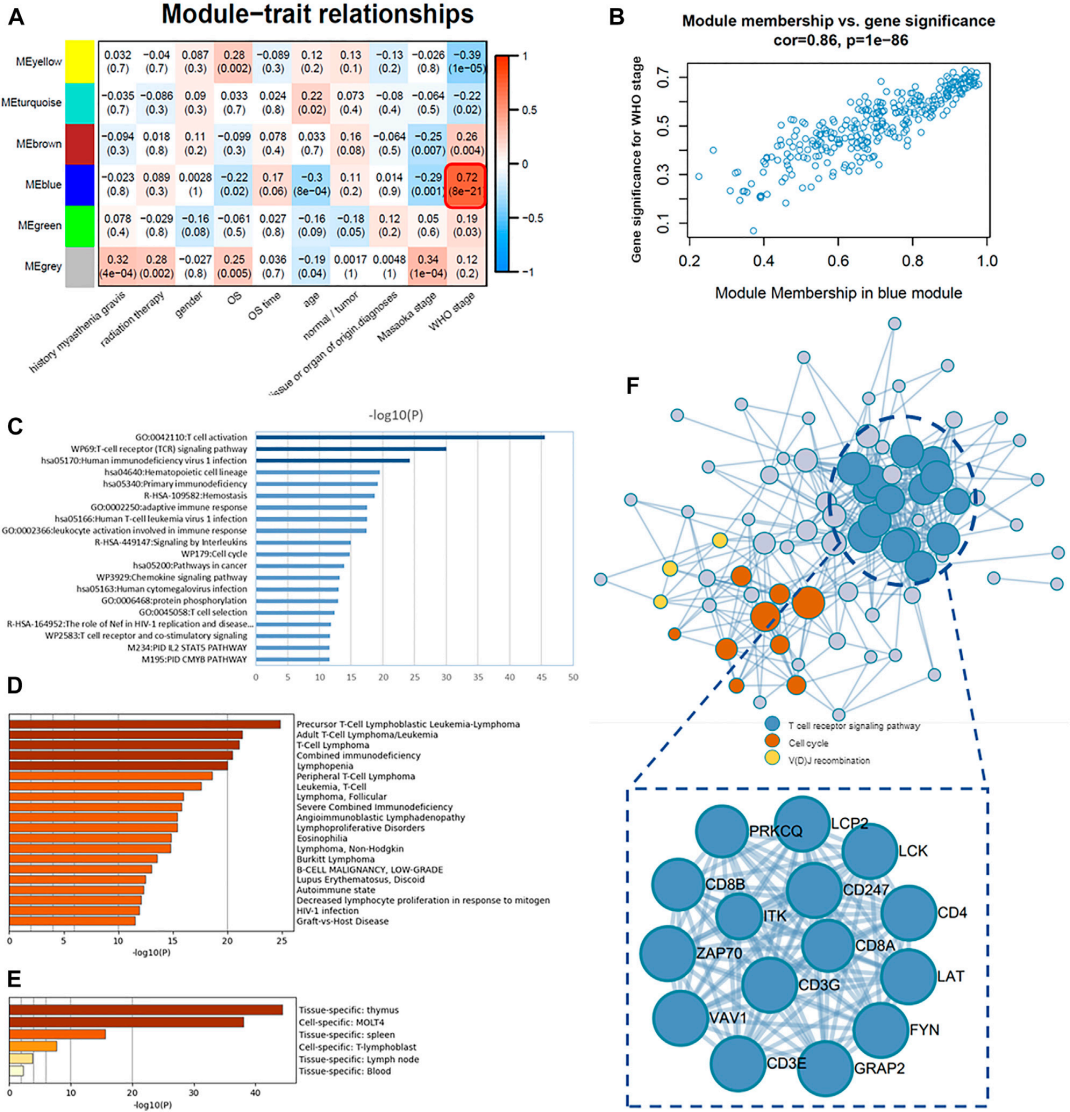
Association of modules with clinical traits and determination of core genes. **(A)** Module–trait associations: each row corresponds to a module eigengene and each column to a trait. Each cell contains the corresponding correlation and *p*-value. **(B)** Scatter plots of GS score and MM for genes in the blue module. **(C)** Pathway and process enrichment analysis: bar graph of enriched terms across 87 core genes (intramodular connectivity >0.7) in the blue module, colored by *p*-values. **(D)** Summary of enrichment analysis in DisGeNET, colored by *p*-values. **(E)** Summary of enrichment analysis in PaGenBase, colored by *p*-values. **(F)** Protein–protein interaction (PPI) network of 87 core genes.

In addition, we calculated the correlation between module genes and clinical traits of thymoma. For each module, the gene co-expression was summarized by the eigengene, and the correlations of each eigengene with clinical traits were calculated, such as history myasthenia gravis, radiation therapy, gender, OS, OS time, age, tumor, tissue or organ, Masaoka stage, and WHO stage. We found that the blue module had the highest correlation with the WHO stage (cor = 0.72, p = 8e−21) (Figures 5A,B).

Therefore, the blue module was analyzed for core genes. The first 87 intersection genes of the blue module with the highest correlation (intramodular connectivity >0.7) were selected as the core genes for subsequent study and enrichment analysis. For details of the core gene list, cf. Table 4.XLSX of the Supplementary Material. The results of functional enrichment analysis were obtained by the online database Metascape; terms with a *p*-value < 0.01, a minimum count of 3, and an enrichment factor >1.5 (the enrichment factor is the ratio between the observed counts and the counts expected by chance) were collected and grouped into clusters based on their membership similarities. We selected the top 20 clusters with their representative enriched terms, mainly involving T-cell activation (GO:0042110), T-cell receptor (TCR) signaling pathway (WP69), human immunodeficiency virus 1 infection (hsa05170), hematopoietic cell lineage (hsa04640), primary immunodeficiency (hsa05340), hemostasis (R-HSA-109582), adaptive immune response (GO:0002250), human T-cell leukemia virus 1 infection (hsa05166), and leukocyte activation involved in immune response (GO:0002366) (Figure 5C). Metascape combines DisGeNET, a comprehensive platform integrating information on human disease–associated genes and variants, and gives the disease information related to these core genes (Figure 5D), most of which are autoimmune diseases or T-cell-related diseases, such as precursor T-Cell lymphoblastic leukemia-lymphoma, T-cell lymphoma, combined immunodeficiency, and lupus erythematosus. Metascape also gives a summary of enrichment analysis in PaGenBase. PaGenBase is a novel repository for the collection of tissue- and time-specific pattern genes. The results show that most of these core genes were specifically expressed in the thymus (Figure 5E), which proved the correctness of these core genes to some extent.

At the same time, the protein–protein interaction (PPI) network of core genes was given by Metascape (Figure 5F), which applied a mature complex identification algorithm called MCODE to automatically extract protein complexes embedded in such a large network. Where the T-cell receptor signaling pathway was the core cluster in this PPI network  (log10(p) = −37.4) , the hub genes of this cluster include CD247, CD8A, CD8B, PRKCQ, CD3E, CD3G, GRAP2, VAV1, CD4, LCK, ZAP70, LCP2, ITK, FYN, and LAT.

### Validation of Hub Genes in the Expression Level

**FIGURE 6:**
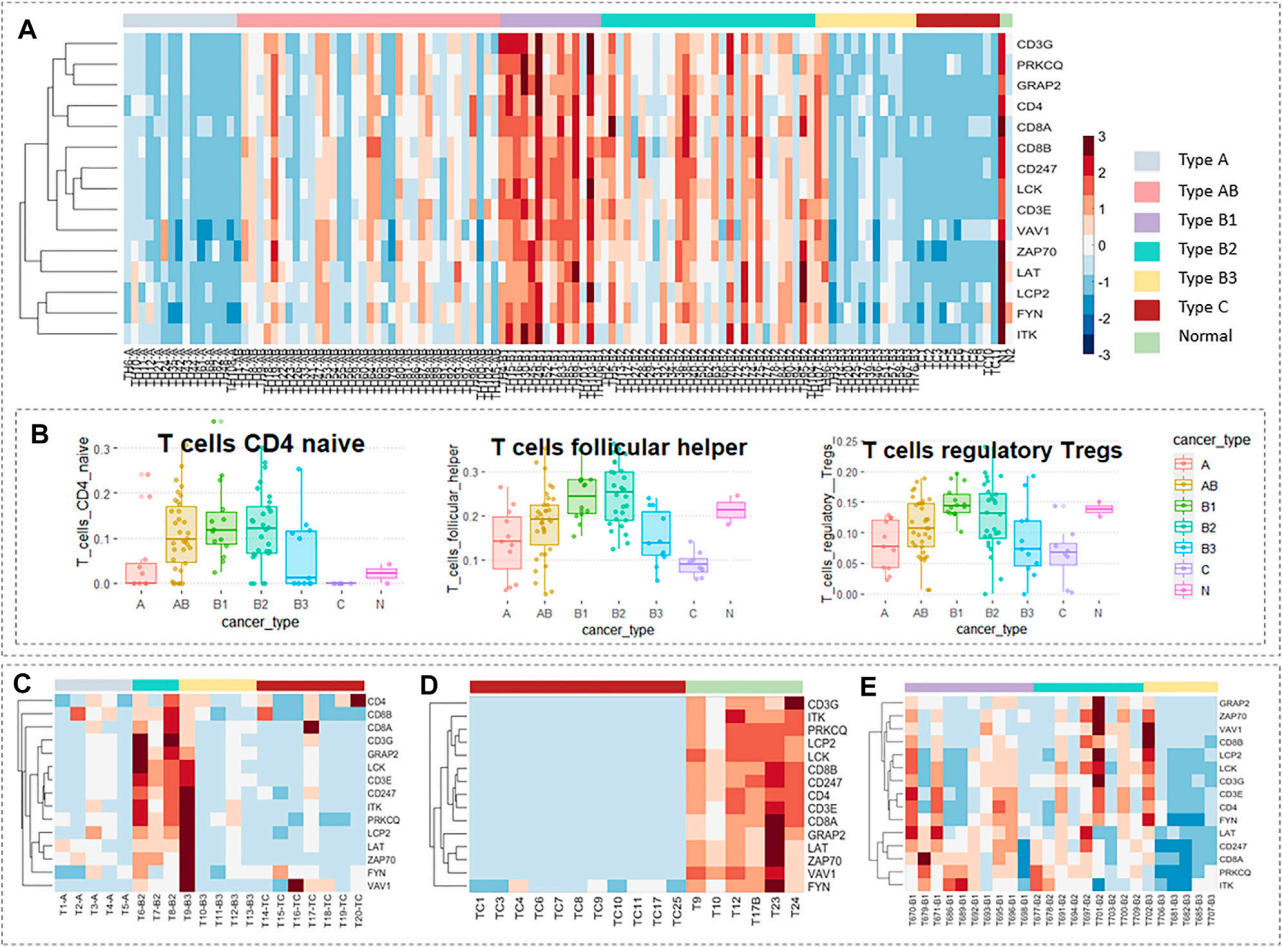
Validation of hub genes in the expression level. Expression levels of 15 hub genes in TCGA data. **(B)** The proportion of T-cells of each thymic tumor subtype in TCGA data obtained by the CIBERSORT algorithm. **(C)** Expression levels of 15 hub genes in GSE57892 data. There are 25 samples in total, including two cases of type AB thymoma, five of type A thymoma, three of type B2 thymoma, five of type B3 thymoma, seven of thymic carcinomas, and three of cell line. **(D)** Expression levels of 15 hub genes in GSE177522 data. There are 19 samples in total, including 11 cases of thymic carcinoids, two cases of thymoma, and six cases of the normal thymus. **(E)** Expression levels of 15 hub genes in GSE29695 data. There are 41 samples in total, including nine cases of type AB thymoma, one of type A thymoma, 10 of type B1 thymoma, nine of type B2 thymoma, six of type B3 thymoma, one of type A/B thymoma, one of type B1/B2 thymoma, and four of the cell line. In particular, cell lines and subtypes of less than three samples were not included in the study.

In the 87 core genes screened by WGCNA, which have a strong correlation with WHO’s classification of thymomas, a core cluster, the T-cell receptor signaling pathway, was obtained by PPI enrichment analysis, which contained 15 interrelated hub genes. We observed the expression levels of these 15 genes in TCGA data using a thermal map (Figure 6A), and found that these hub genes that were lowly expressed in type A and type B3 thymomas and thymic carcinomas are highly expressed in type B1 and type B2 thymomas. There is no consistent expression pattern in AB thymomas, but thymoma patients with MG have relatively low-expression in AB (cf. SI-2 of the Data Sheet 1.PDF). To verify these results, three groups of thymic tumor data were used as validation data, and the data processing method was consistent with TCGA data, which were standardized. In GSE57892, type A and B3 thymoma and thymic carcinoma has almost low expression in these 15 hub genes, while type B2 thymoma has a large expression level (Figure 6C). The data GSE177522 also verify the result that the 15 hub genes were almost low expressed in thymic carcinoma (Figure 6D). The data GSE29695 clearly show that B3 thymoma cannot be expressed in these 15 hub genes, and the relative expression levels of B1 and B2 were higher (Figure 6E).

The WHO stage is mainly based on the morphology of thymoma epithelial cells and the ratio of lymphocytes to epithelial cells in tissues. We know that these 15 genes which have a strong correlation with WHO classification belong to the T-cell receptor signaling pathway. Interestingly, the variation trend of the proportions of three T-cells in different thymic tumor subtypes obtained from the CIBERSORT algorithm in Figure 6B was very consistent with the variation trend of the expression levels of these 15 hub genes (cf. Table 2.XLSX of the Supplementary Materials). A similar trend of expression spectrum and immune infiltration can be used as an inspiration for the phenomenological hypothesis in this group of hub genes.

Certainly, for sake of comprehending whether the 15 hub genes are related to the prognosis of patients, we performed a Kaplan–Meier analysis of these genes according to the clinical data of 119 cases of thymomas in TCGA. It was found that 14 genes were associated with prognosis except FYN ( p < 0.05 ). The Kaplan–Meier survival curves of 15 hub genes are given in SI-6 of the Data Sheet 1.PDF.

Taken together, these validation analyses confirm that the more severe the subtype of thymomas is, the less the number of T-cells is, and the expression of related genes in the T-cell receptor signaling pathway is defective.

## Discussion

In this study, we obtained 14 hub immune genes of thymomas through statistical analysis. The common correlation pathway of these 14 genes was the T-cell receptor signaling pathway, and their expression was closely related to the WHO stage.

It is well known that there are generally no specific symptoms for patients with thymomas, but they may have nonspecific symptoms such as chest pain, chest tightness, palpitation, fatigue, and cough. Therefore, they are often ignored by patients who miss the best opportunity for early diagnosis. The first diagnosis is usually found by chance in physical examination or because of the physical discomfort caused by PNS. Thymoma is closely related to autoimmune disorders, and most of its concomitant PNS are autoimmune diseases. Therefore, in this study, we selected two kinds of autoimmune PNS, namely, systemic lupus erythematosus (GSE138458) and rheumatoid arthritis (GSE93272), as the objects of discussion. At the same time, specific pulmonary fibrosis (GSE33566), a severe complication that often occurs after thymic tumor surgery, was also selected. There were 330 samples in dataset GSE138458, including 307 cases of systemic lupus erythematosus (SLE) and 23 cases of healthy control. For dataset GSE93272, there were 232 cases of rheumatoid arthritis (RA) and 43 cases of healthy control. The dataset GSE33566 includes 93 cases of idiopathic pulmonary fibrosis (IPF) and 30 cases of healthy control.

**FIGURE 7:**
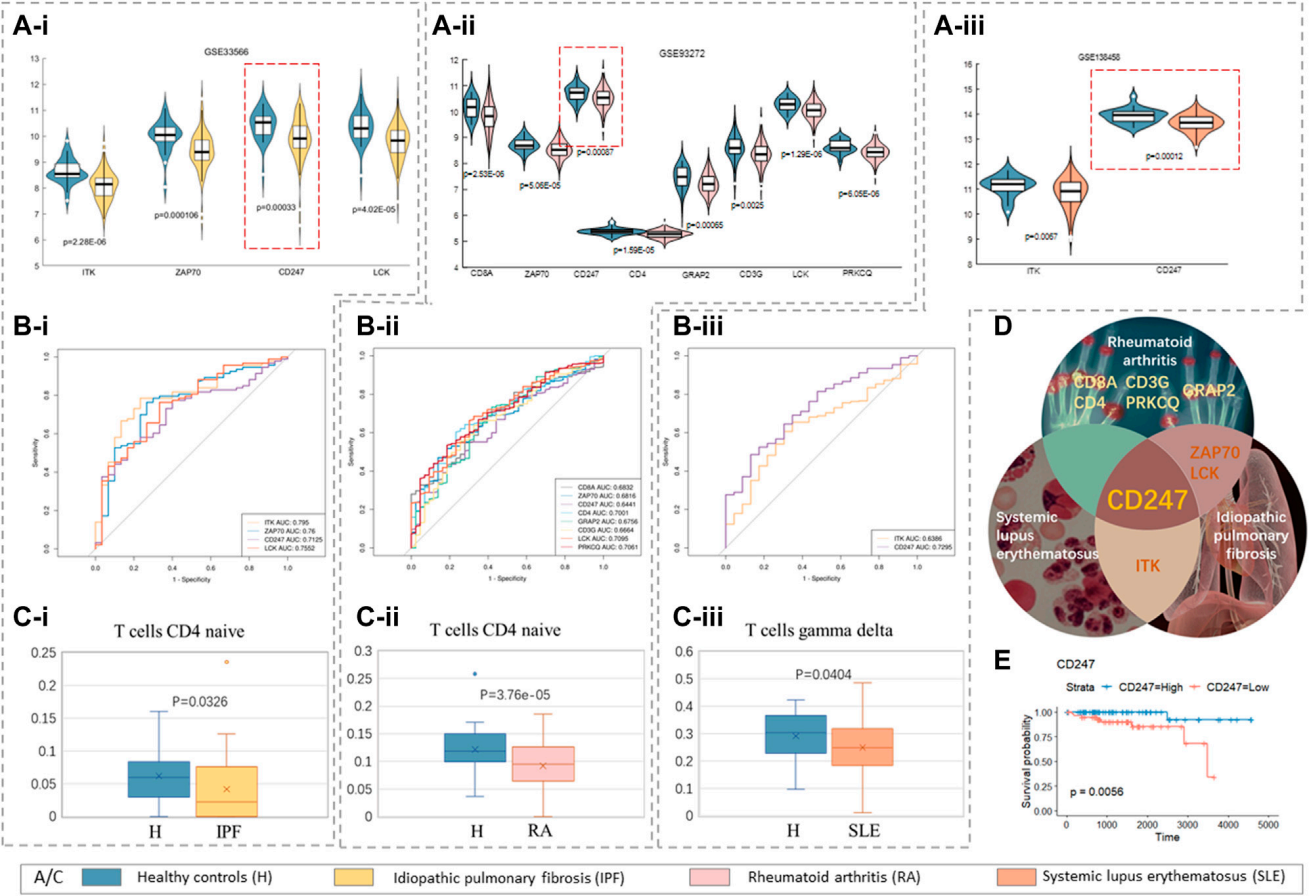
Paraneoplastic syndrome. **(A–i)** Idiopathic pulmonary fibrosis, GSE33566; **(A–ii)** rheumatoid arthritis, GSE93272; **(Aiii)** systemic lupus erythematosus, GSE138458; **(A–i,ii,iii)** violin maps of hub genes with significant differences in expression in three PNS. **(B–i,ii,iii)** The area under the curve of these significant genes (AUC) by receiver operating characteristics (ROC) analysis. **(C–i,ii,iii)** The box patterns of T-cells with significant differences in the proportions of three PNS. **(D)** Outline map of intersection genes of three PNS (cf. Table 2.XLSX of the Supplementary Materials). **(E)** The Kaplan–Meier survival curves of CD247.

Considering the condition that the *p*-value of survival analysis was less than 0.01 and the *p*-value of expression difference was less than 0.01, we found that ITK, ZAP70, CD247, and LCK were lower expressed in patients with specific pulmonary fibrosis than in healthy controls (Figure 7A–i). The expression of CD8 A, ZAP70, CD247, CD4, GRAP2, CD3 G, LCK, and PRKCQ in patients with rheumatoid arthritis was lower than that in healthy controls (Figure 7A–ii). Compared with the healthy control group, the expression levels of ITK and CD247 in patients with systemic lupus erythematosus decreases (Figure 7A–iii). All hub genes expression levels were significantly different between the highest and lowest quartiles, and the area under the curve (AUCs) of these genes was higher than 0.6 (Figures 7B–i,ii,iii), confirming the authenticity of the differences in these genes in their respective PNS. We calculated the proportion of immune cells in patients with three types of by CIBERSORT concomitantly, and found that the proportion of T-cells that were CD4 naive in patients with specific pulmonary fibrosis and rheumatoid arthritis was significantly different, while the number of T-cells gamma delta in patients with systemic lupus erythematosus was significantly different.

In summary, the decreased proportion of T-cells and the lack of hub gene expression are the common links between thymomas and specific pulmonary fibrosis, rheumatoid arthritis, and systemic lupus erythematosus. The lack of expression of different core genes may be the reason why different patients tend to have different PNS.

Obviously, CD247 was at a low expression level in all three PNS (Figure 7D), and the Kaplan–Meier survival curves of CD247 in TCGA data ( p = 0.0056 ) were given in (Figure 7E). The protein encoded by CD247 (also called CD3ζ) is T-cell receptor zeta. The zeta chain plays an important role in coupling antigen recognition to several intracellular signal-transduction pathways. Low expression of the antigen results in impaired immune response (Call et al., 2006). Moreover, this gene plays an important role in intrathymic T-cell differentiation, and its lack of expression may lead to the reduction of mature T-cells. Petros Christopoulos et al. (2015) proposed a novel thymoma-associated immunodeficiency in 2015. Its characteristics are an accumulation of CD247-deficient, hyporesponsive naive γδ and αβ T-cells and an increased susceptibility to infections (Christopoulos et al., 2015). In 2018, his team further suggested that deficient CD247 expression was a typical histopathological characteristic of thymomas with cortical features (Christopoulos et al., 2018). Recent evidence has demonstrated that CD247 is a potential T-cell–derived disease severity and prognostic biomarker in patients with idiopathic pulmonary fibrosis (Li et al., 2021). Some studies have shown that CD3ζ plays a vital role in multiple autoimmune diseases, such as the gene expression assays showing that CD3ζ mRNA levels are downregulated in PBMCs of patients with RA when compared to healthy controls (Li et al., 2016). Moreover, available evidence suggests that SLE is associated with a deficiency in a cluster of differentiation 247 (Takeuchi and Suzuki, 2013). CD247 is shared by various autoimmune disorders and supports a common T-cell–mediated mechanism. A classic T-cell phenotype in SLE is the downregulation and replacement of the CD3ζ chain that alters T-cell receptor signaling (Martins et al., 2015).

In summary, the common characteristics of thymomas and these three PNS are the low expression of CD247 and the inhibition of T-cell differentiation.

## Conclusion

In this study, 14 hub immune genes closely related to thymomas, jointly involved in the T-cell receptor signaling pathway, were found by analysis of the expression data of immune genes in thymomas. Compared with normal thymus and type B1/ B2 thymoma, the number of T-cells in type A/B3 thymoma and thymic carcinoma is less, and the expression of genes related to T-cell receptor signaling pathway is low. Then, we also discussed the expression of these 14 core genes in three PNS, and found that CD247 has not only minimal expression in multiple subtypes of thymomas, but also low expression in specific pulmonary fibrosis, rheumatoid arthritis, and systemic lupus erythematosus. The low expression of CD247 and the decrease in the number of mature T-cells are the common features of thymomas and these three PNS.

## Data Availability

Publicly available datasets were analyzed in this study. These data can be found here: TCGA-THYM https://portal.gdc.cancer.gov/ GSE177522, GSE57892, GSE29695, GSE93272, GSE138458, and GSE33566 https://www.ncbi.nlm.nih.gov/geo/.
