# Supplementary material for: Identification of Immune-Related Hub Genes in Thymoma: Defects in CD247 and Characteristics of Paraneoplastic Syndrome
Source: Front Genet. 2022 Jun 14;13:895587. doi: 10.3389/fgene.2022.895587 (PMC9237438; doi:10.3389/fgene.2022.895587)
Supplement: Supplementary file 3 [file DataSheet1.PDF]

SI-1 Variance and Spearman's rho

Filtering the genes by variance is a common method in the literature [1]. Variance

$$Var(X) = E\left[\left(x_i - \overline{x}_i\right)^2\right]$$

measures the error of a single gene, and covariance is used in probability theory and statistics to measure the correlation of two variables. Variance is a special case of covariance when two variables are the same. In this work we remove the noisy genes by covariance. The algorithm of Spearman's rho employs the covariance. Compared with the variance method, the information contained in noise genes screened by Spearman's rho is more diverse (SI_Fig 1). It shows that the Spearman's rho has a better global reach.

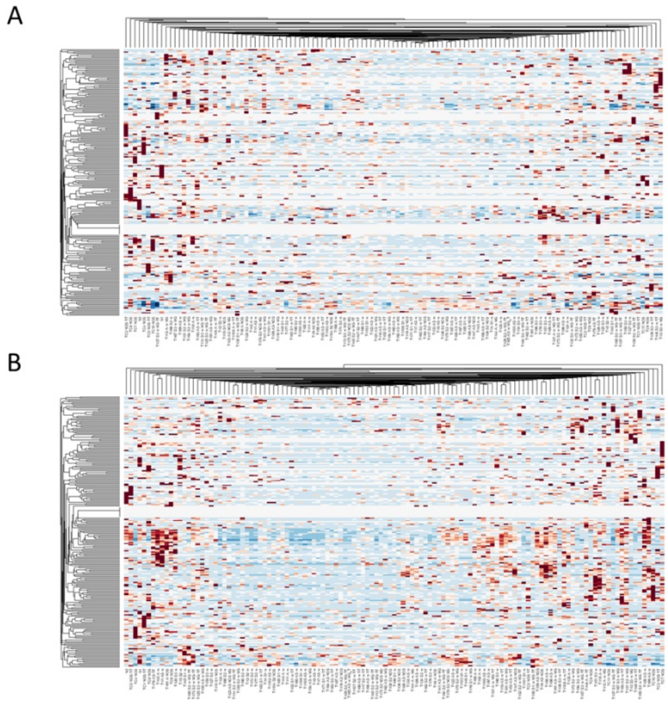

SI_Fig 1. Variance and Spearman's rho. (A) Noise genes eliminated by correlation coefficient (224 genes). (B, D) Noise genes eliminated by variance (224 genes).

Spearman's rho is equivalent to Pearson's Linear Correlation Coefficient applied to the rankings of the columns x_a and y_b [2]. Pearson's linear correlation coefficient rho(a,b) is defined as:

$$rho_{pearson}(a,b)=\frac{\sum_{i=1}^{n}\left(x_{a,i}-\overline{x}_a\right)\left(y_{b,i}-\overline{y}_b\right)}{\left[\sum_{i=1}^{n}\left(x_{a,i}-\overline{x}_a\right)^2\sum_{i=1}^{n}\left(y_{b,i}-\overline{y}_b\right)^2\right]^{1/2}}$$

where n is the length of each column. If all the ranks in each column are distinct, the equation simplifies to:

$$rho_{spearman}(a,b)=1-\frac{6\sum d^2}{n\left(n^2-1\right)}$$

where d is the difference between the ranks of the two columns, and n is the length of each column. Values of the correlation coefficient can range from –1 to +1. A value of –1 indicates perfect negative correlation, while a value of +1 indicates perfect positive correlation. A value of 0 indicates no correlation between the columns. It is determined that the two genes are not correlated when the correlation coefficient r is in the range of (− 0.2, 0.2). When a gene is not correlated with 70 % of the remaining genes, it is determined to be a noise gene.

The code is as follows:

```
[R2,P2]=corr(data','type','Spearman');
hcor2=clustergram(R2,'columnlabels',gene,'rowlabels',gene,'displayratio',[0.1,0.1],'Colormap',
redbluecmap);
for i=1:m
    G(i,:) = sum(sum(R2(i,:)>-0.2 & R2(i,:)<0.2));
end
F = find(G<m*0.7);
data2 = data(F,:);
gene2 = gene(F);
```

[1] Weifen Zhu, Ziming Zhang, Weiwei Gui, et al, Identification of the Key Pathways and Genes in Hypoxia Pulmonary Arterial Hypertension Following Intrauterine Growth Retardation, Front Mol Biosci. 2022 Mar 31;9:789736. doi: 10.3389/fmolb.2022.789736.

[2] Best, D.J., and D.E. Roberts. "Algorithm AS 89: The Upper Tail Probabilities of Spearman's rho." Applied Statistics, 24:377-379.

SI-2 PNS- myasthenia gravis

Myasthenia gravis (MG) is the most common paraneoplastic syndrome of thymoma., a sizeable percentage (25–40%) of thymoma patients with MG. There are 34 patients complicated with MG among 119 thymoma patients in TCGA data set. Meanwhile, one of the two normal samples has a history of MG. The expression levels of 15 genes in thymoma patients with MG were observed (SI_Fig 2). In type A and B, it was found that the expressions of patients with MG were consistent with those without MG. However, patients with AB type are different, and thymoma patients with MG basically have low expression in AB

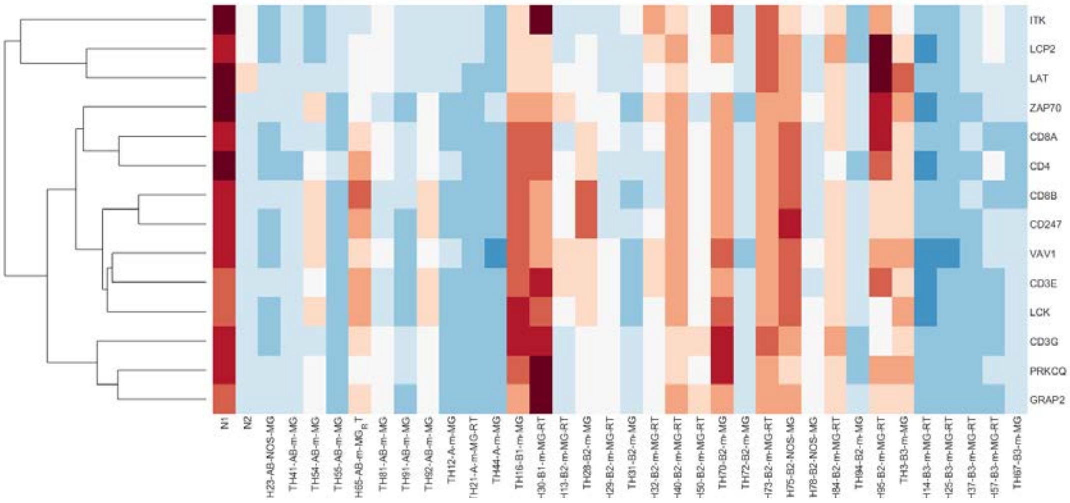

SI_Fig 2. myasthenia gravis.

SI-3 supplementary data of the normal samples

This work aims to uncover the core genes closely related to different WHO types. Hence the relatively small number of normal samples should not become a major concern. There are two normal samples in TCGA dataset, one from the thymus tissue of normal people, but the other from the thymus tissue of patients with myasthenia gravis. Therefore technically, only one normal sample can be used. We agree that it is important to include sufficient normal samples in cancer research. Therefore, we further selected the dataset GSE177522, containing 6 normal thymus samples, as the supplementary data of the normal samples.

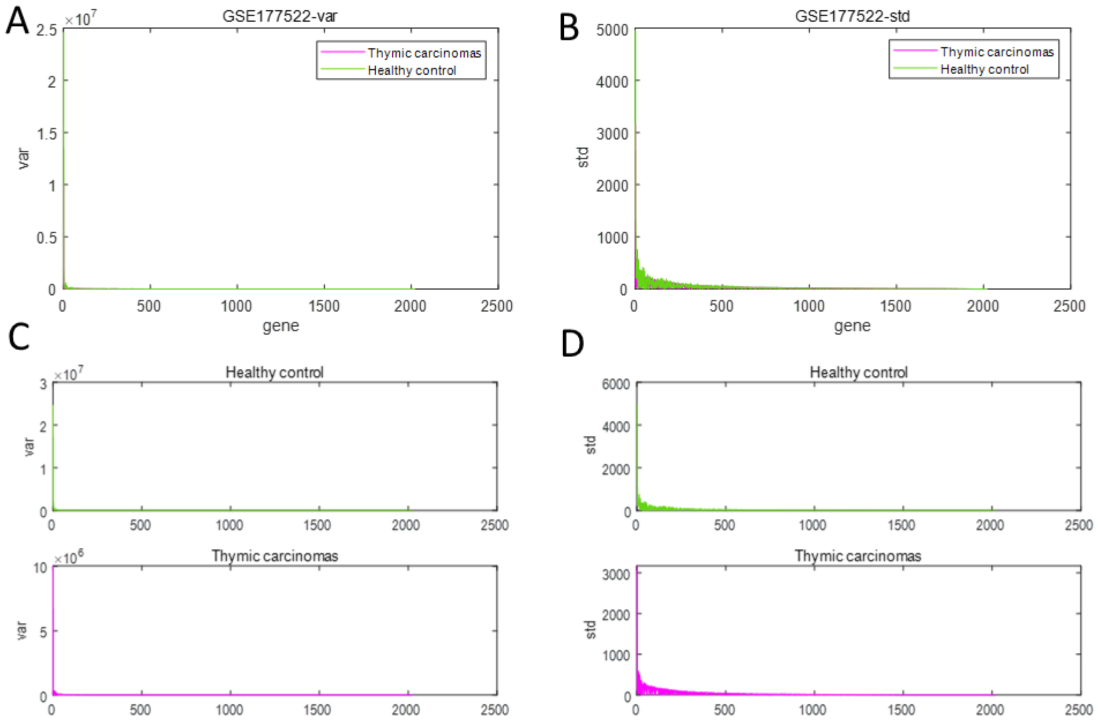

SI_Fig 3. Variance and standard deviation of 2023 immune genes (GSE177522). (A, C) Variance of 2023 immune genes in healthy controls and thymic carcinomas. (B, D) Standard deviation of 2023 immune genes in healthy controls and thymic carcinomas.

We validated the variance and standard deviation of each group of the data to assess the within-group dispersion (SI_Fig 3). For all the immune genes used in this study, the variance and standard deviation of most genes in the thymus tumor and healthy control group are at a low level, which indicates a low dispersion of samples within the group. In addition, the variance

and standard deviation of most genes in thymus tumor and healthy control group are similar. In other words, The data selection is consistent with general statistical rules in these limited samples.

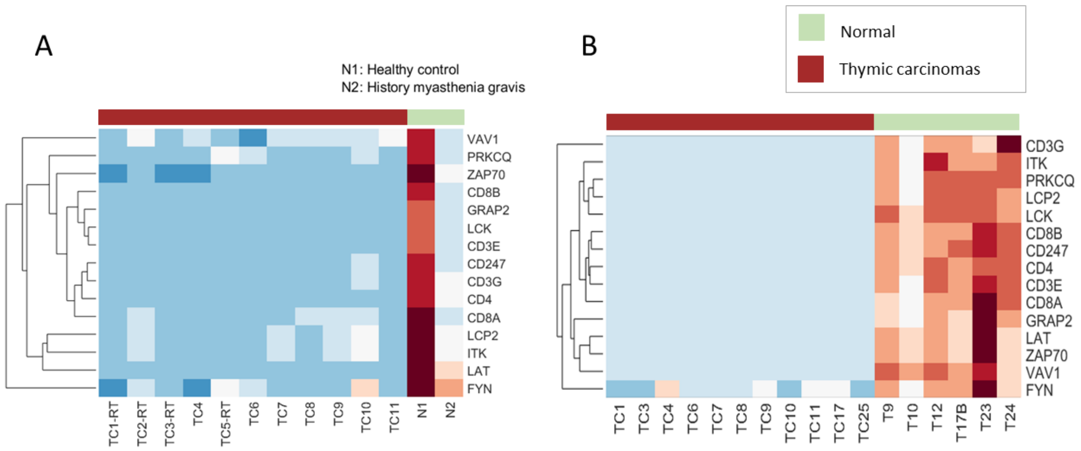

SI_Fig 4. Validation of hub genes in the expression level. (A) TCGA: Expression levels of hub genes in thymic carcinomas and normal thymus tissues. Particularly, N1 comes from the thymus tissue of normal people, and N2 comes from the thymus tissue of patients with myasthenia gravis. (B) GSE177522: Expression levels of hub genes in thymic carcinomas and normal thymus tissues.

We compared the expression levels of 15 hub genes in TCGA data set and GSE177522 data set between thoracic adenocarcinoma samples and healthy control samples, and found that the expression patterns of all hub genes were similar in both groups. (SI_Fig 4). In particular, N2 is not seen as a normal sample.

Based on the above discussion, we conclude that GSE177522 dataset can be used as the supplementary data to the normal samples.

SI-4 variance and standard deviation of all immune genes

Similarly, for TCGA data, the variance and standard deviation of all immune genes in each thymoma group were observed. (SI_Fig 5). Most genes have low dispersion within groups and similar variance and standard deviation between groups (SI_Fig 5 A-D). In addition, the intra-group correlation coefficient of each thymoma is close to 1, i.e., the samples within the group are highly similar (SI_Fig 5 E).

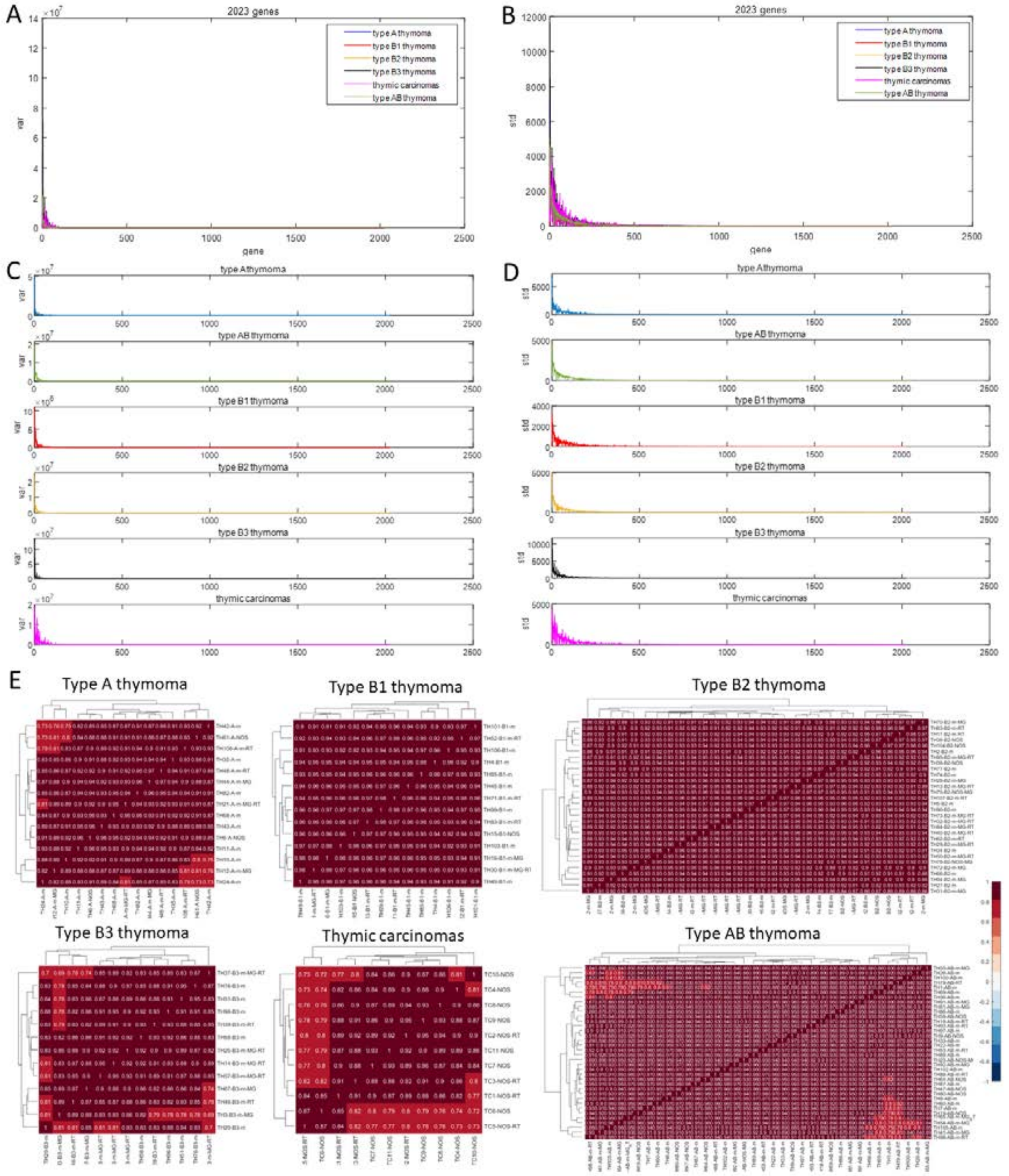

SI_Fig 5 Variance and standard deviation of 2023 immune genes (TCGA). (A, C) Variance of 2023 immune genes in type A / AB / B1 / B2 / B3 thymoma and thymic carcinomas. (B, D) Standard deviation of 2023 immune genes in type A/AB/B1/B2/B3 thymoma and thymic carcinomas. (E) Heat map of correlation coefficient of intra-group samples.

In summary, the data selection is consistent with general statistical rules. First, we found the dataset GSE177522 as a supplement to the normal sample, and verified its reliability by variance and expression level. Second, in the TCGA dataset, although the number of samples for each thymoma is different, the intra-group dispersion is small and the sample similarity is rather high. They show roughly the same variance and standard deviation between groups are close, which ought to have reasonably justified the authenticity of the data

SI-5 Relationship between expression profile and immune infiltration

The change trend of expression profile of hub gene in several thymomas is similar to that of the proportion of three T cells, it may give rise to an illusion of low gene expression and low survival rate. The focus of this work is to explore the hub genes related to the WHO stage and explore their relationship with paraneoplastic syndrome of thymoma. Note that immune infiltration echos the expression level of hub genes, which is not the focus of this work. Though the gene expression level of T cell receptor signaling pathways is related to T cell immune infiltration, but their relationship is nonlinear. We have demonstrated the non-linearity in this work (SI_Fig 6).

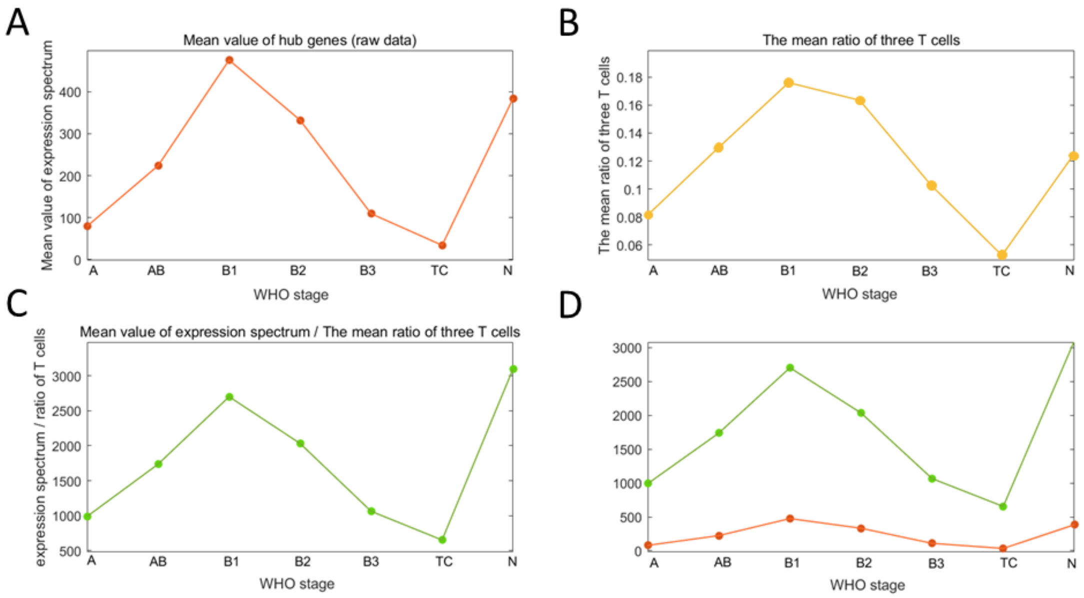

SI_Fig 6 The relationship between expression profile and immune infiltration (TCGA). (A) mean expression level of hub genes in type A / AB / B1 / B2 / B3 thymoma, thymic carcinomas and normal samples. (B) The mean ratio of three T cells (T cells CD4 naïve, T cells follicular helper, T cells regulatory Tregs). (C) mean expression level of hub genes divided by mean ratio of T cells. (D) Two curves of figure A and figure C.

The variation trend of the proportions of three T cells in different thymic tumor subtypes obtained from Cibersort algorithm is very consistent with the variation trend of the expression levels of 15 hub genes (SI_Fig 6 A, B). The results obtained by dividing the two are still in a similar trend, which indicates that the relationship between them is nonlinear. Therefore the similarity in the trend of

expression spectrum and immune infiltration can be seen as as an inspiration to the phenomenological hypothesis for this group of hub genes.

SI-6 The Kaplan–Meier survival curves of 15 hub genes.

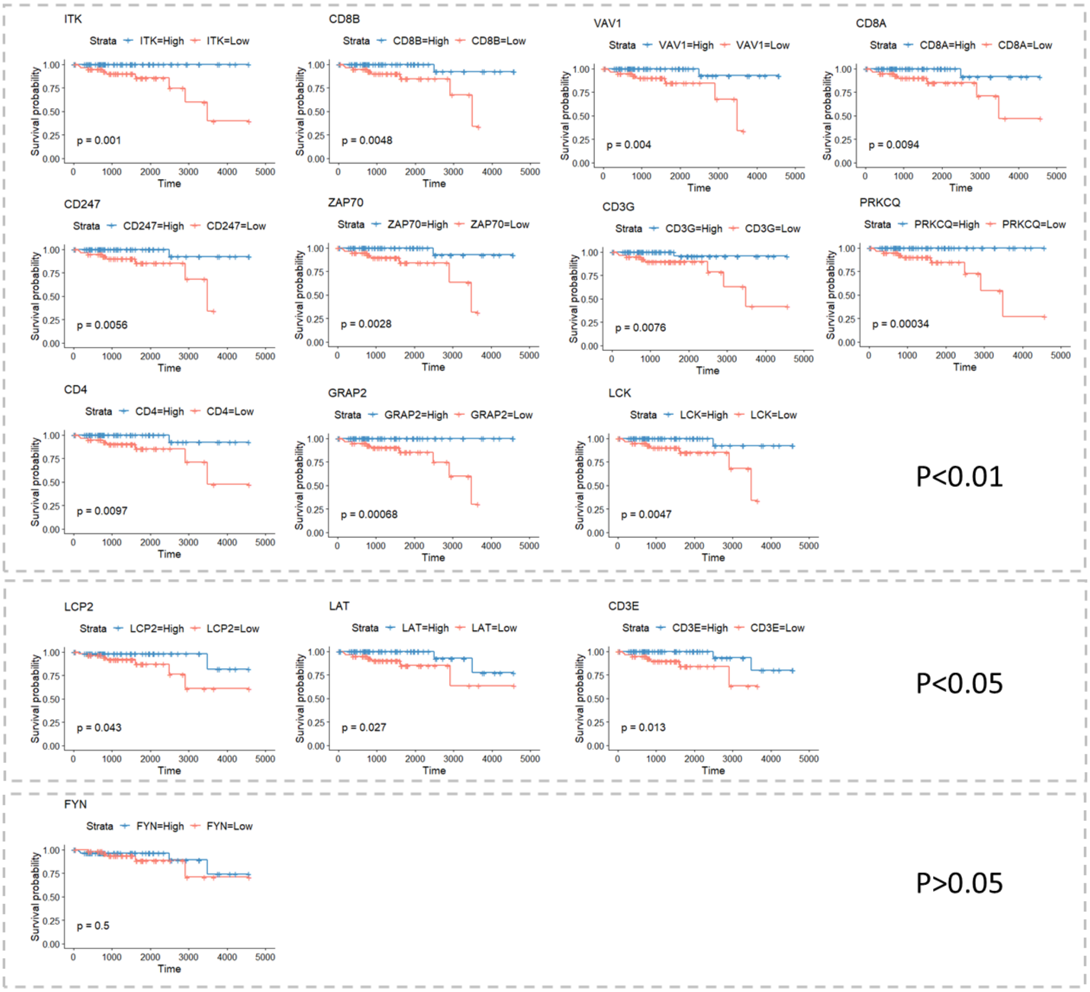

SI_Fig 6. The Kaplan–Meier survival curves of 15 hub genes.
